# Swedish population data for 37 autosomal, 12 X-chromosomal and 23 Y-chromosomal STR loci

**DOI:** 10.1007/s00414-026-03793-2

**Published:** 2026-04-09

**Authors:** Adam Staadig, Ronny Hedell, Arvid Hedén Gynnå, Ricky Ansell, Andreas Tillmar

**Affiliations:** 1 Department of Forensic Genetics and Forensic Toxicology, National Board of Forensic Medicine, Artillerigatan 12, Linköping, SE 587 58 Sweden; 2 Department of Biomedical and Clinical Sciences, Faculty of Health Sciences, Linköping University, Linköping, SE 581 83 Sweden; 3 National Forensic Centre, Swedish Police Authority, Linköping, SE 581 94 Sweden; 4 Department of Physics, Chemistry and Biology, Linköping University, Linköping, SE-581 83 Sweden

**Keywords:** Population genetics, STR, DNA sequencing, Allele frequencies, Haplotype frequencies, Swedish population

## Abstract

Population genetic analysis is a fundamental component in forensic genetics for ensuring the reliability and validity of DNA evidence interpretation. Here, we present a comprehensive forensic genetic analysis of a contemporary Swedish population, based on 37 autosomal, 12 X-chromosomal and 23 Y-chromosomal short tandem repeat (STR) loci. Blood samples were systematically collected from individuals across all 21 counties of Sweden. Five commercial forensic STR kits (PowerPlex Fusion 6C System, PowerPlex Y23 System, Investigator HDplex kit, Investigator X12QS kit and Mainstay SE) were analysed. In total, autosomal STR allele frequencies were generated from 538 individuals, and X- and Y-chromosomal haplotypes were established from 513 males. High genotype concordance (> 99.95%) was observed across overlapping loci between kits, confirming the robustness and reliability of the investigated systems. A few discrepancies were identified, primarily due to STR nomenclature differences between capillary electrophoresis and sequencing generated alleles. Both fragment length-based and sequence-based allele frequencies were obtained. Statistical analysis revealed high discriminatory power, no signs of deviations from Hardy-Weinberg equilibrium, and no evidence of linkage disequilibrium between any pair of loci. Minimal genetic differentiation was observed within Sweden and between Sweden and geographically close populations, whereas significant genetic differences were found when comparing with more distant populations. These findings confirm that the analysed STR loci are well suited for forensic applications in the Swedish population. The resulting allele and haplotype frequency data establish an updated and expanded population-specific reference database, which is expected to serve as a valuable resource for forensic casework in Sweden.

**Supplementary Information:**

The online version contains supplementary material available at 10.1007/s00414-026-03793-2.

## Highlights

An updated and expanded STR reference database for the Swedish population.

Comprehensive concordance analysis using five forensic STR kits.

Increased allelic diversity when using massively parallel sequencing.

Minor genetic differences within Sweden and between Sweden and geographically close populations.

**Supplementary Information:**

The online version contains supplementary material available at 10.1007/s00414-026-03793-2.

## Introduction

Population genetics plays a central role in forensic science by providing the statistical framework needed for interpreting DNA evidence. Statistics, such as the likelihood ratio (LR), are calculated to estimate the weight of evidence in forensic investigations, and these calculations rely on accurate and representative allele frequency data from relevant reference populations. Improper population data can lead to overestimation or underestimation of the LR [1]. Fragment length analysis of short tandem repeats (STRs) using capillary electrophoresis (CE) has long been the gold standard in forensic DNA analysis worldwide [2]. The number of STR loci included in commercial multiplex kits has grown significantly over the past three decades, from just a few markers in the 1990s [3] to more than twenty loci in kits commonly used today [4]. Moreover, the combination of different kits may be particularly useful in certain kinship cases to increase the discriminatory power [5]. As STR multiplexes expands, updated and population-specific allele frequency data sets are essential to ensure accurate and robust forensic interpretations, particularly when introducing new loci into routine casework.

Massively parallel sequencing (MPS) has been increasingly adopted in forensic genetics in the last decade. Unlike CE, which only measures the length of STR fragments, MPS provides the full nucleotide sequence of STR alleles, revealing additional polymorphisms caused by sequence variation within the repeat region. Additionally, MPS enables analysis of flanking regions adjacent to STR loci, further increasing the genetic information. Several studies have reported the increased genetic information gained with MPS analysis of STRs and its increase in power of discrimination. As a result, sequence-based population genetic studies have been initiated by several forensic laboratories in the recent years [6–13], to support the implementation of MPS.

In Sweden, multiple allele frequency databases are used at the National Board of Forensic Medicine and the Swedish Police Authority, and the remaining volume of original samples and the details of the underlying samples, such as the origin of the individuals, are somewhat limited. In addition, renewed sample collection may be desirable in the light of changing ethical standards. To address these limitations, we initiated a project aimed at creating a new, harmonized and ethically sound population reference database suitable for forensic use across Sweden.

In this study, we systematically collected blood samples from individuals across all twenty-one counties in Sweden. We report updated allele frequencies and relevant forensic parameters from both CE and MPS generated data. This includes autosomal and sex-specific STRs based on five commercially available STR kits. The addition of sequence based STR data will not only support current CE-based analysis but also pave the way for broader adoption of MPS in casework. We also compared our new data set with the existing Swedish reference databases to assess potential shifts or differences in allele distributions. Finally, we investigated intra-population substructure within our dataset and inter-population differences by comparing our results with geographically close as well as distant populations.

## Materials and methods

### Sample collection and selection

**Fig. 1:**
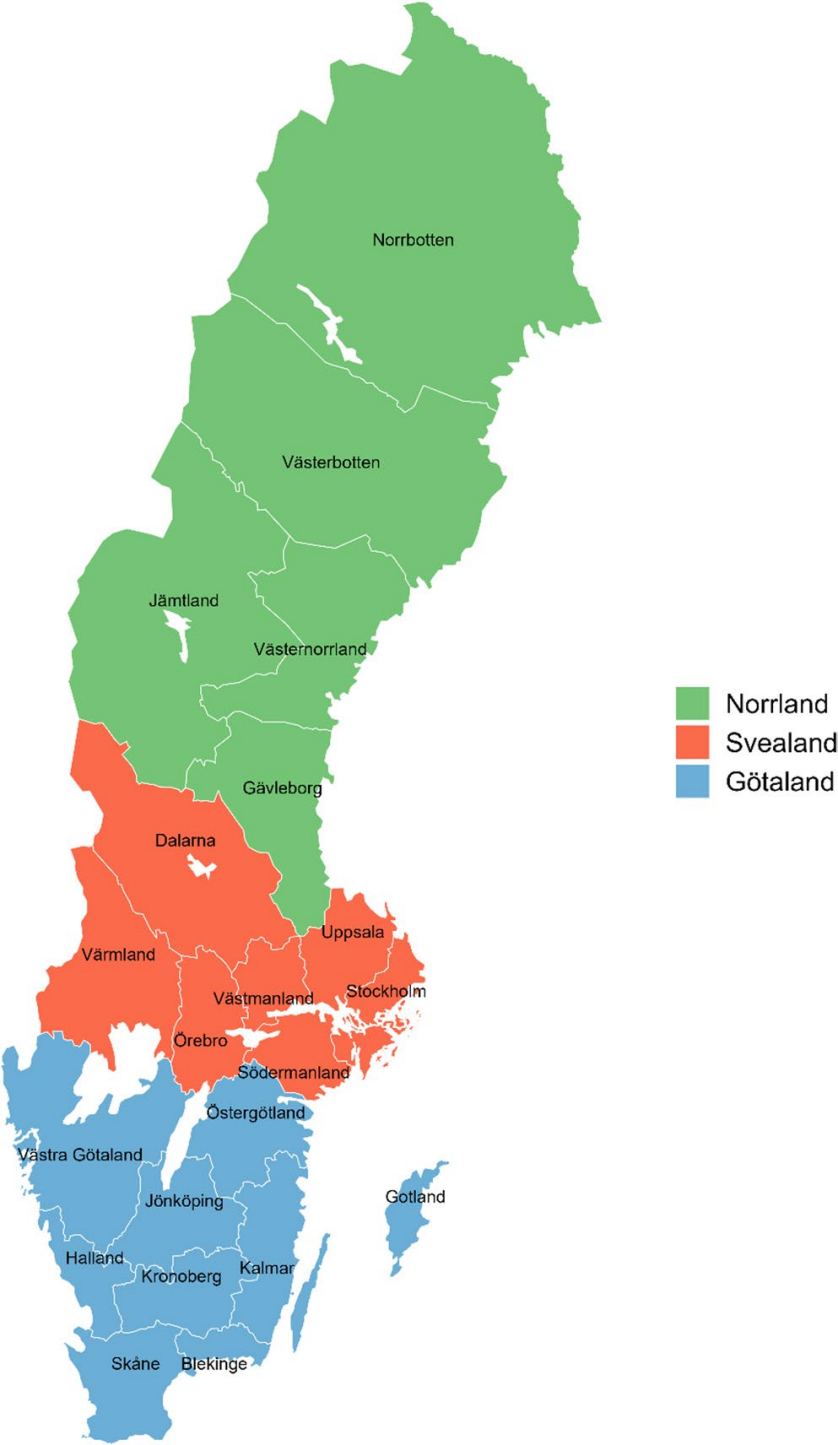
Map of Sweden showing the 21 counties. The three lands of Sweden are indicated in green for Norrland, red for Svealand and blue for Götaland

Samples were collected between 2020 and 2025 from each of the twenty-one counties in Sweden (Swedish “län”, Fig. 1). As healthcare in Sweden is decentralized and administered by regional authorities, each responsible for the healthcare system within its corresponding county, this sampling strategy was applied to efficiently achieve nationwide representation. Blood samples (EDTA) were obtained from two distinct sources: (1) within the Swedish healthcare system (i.e. healthcare centres and hospitals), where individuals provided blood samples in connection with medical evaluations, and (2) blood centres, where healthy individuals voluntarily donated blood as part of routine blood donation. This dual-source strategy was chosen to reduce sampling bias toward either healthy blood donors or hospital visitors, while maintaining a practical and efficient collection approach. A questionnaire was provided during sampling, consisting of ancestry and phenotype relevant questions, including the participants and their grandparent’s country of birth. Additionally, if the participant was born in Sweden, information on their county of birth was included. From all collected samples, 820 samples were selected for this study (71% from the healthcare system and 29% from blood centres) with the inclusion criteria that each individual and their four grandparents were born in Sweden. Furthermore, selection was based on their county of birth resulting in a sample set where the number of samples from each county imitate the percentual distribution of residents in the county (Supplementary Table S1).

Collected blood samples were transferred from the EDTA tubes to two types of DNA collection cards i.e. QIAcard FTA (Whatman, Qiagen, Hilden, Germany) and GenSaver 2.0 (Ahlstrom, Helsinki, Finland). Autosomal STR typing was performed for 552 samples (304 females and 248 males). X- and Y-STR typing was done for 516 males, enabling haplotyping. Theses samples consisted of all 248 male individuals from the 552-sample set used for autosomal typing, and 268 additional males.

### Fragment length-based STR typing

Samples were analysed using four CE-based STR typing kits: PowerPlex Fusion 6C System (Promega Corporation, Madison, WI, USA) [14], Investigator HDplex Kit (Qiagen, Hilden, Germany) [15], Investigator Argus X-12 QS Kit (Qiagen) [16, 17] and PowerPlex Y23 System (Promega Corporation) [18]. Details of the included STR markers in each analysed kit is described in Supplementary Table S2. All kits were analysed in accordance with the manufacturer’s protocols, with the following exceptions. The number of PCR cycles was modified for the Investigator HDplex kit, Investigator Argus X12 QS kit, and PowerPlex 6C System (PPF6C) which were set to 29, 24, and 27 cycles, respectively. The volume of PCR mix for PPF6C was 25 µl. The loading cocktail for CE was 0.6 µl WEN ILS 500, 0.6 µl Stabilizer Reagent (DM6571, Promega Corporation), and 11.5 µl HiDi formamide for PPF6C. These adjustments were made based on findings during internal validation studies (data not shown). For the PPF6C, direct amplification of GenSaver 2.0 card punches (1.2 mm) was performed using a HID VeritiPro Thermal Cycler (Thermo Fisher Scientific, Waltham, MA, USA). STR fragments were separated and detected using an ABI3500xL Genetic Analyzer with 36 cm capillary array and POP-4 polymer, with injection parameters set to 24 s at 1.2 kV (Thermo Fisher Scientific). Data analysis was carried out using 3500 Series Data Collection Software v.4 and GeneMapper ID-X version 1.6 (Thermo Fisher Scientific). The following analytical thresholds (RFU) were applied for typing an allele: blue: 120, green: 155, yellow: 85, red: 115 and purple: 60, together with a global cut-off value at 15%, all derived from internal validation. QIAcard FTA collection card punches (1.2 mm) was used for all other STR kits. The Investigator HDplex, Investigator Argus X12 QS and PowerPlex Y23 kits were amplified on a Veriti Thermal Cycler (Thermo Fisher Scientific). The amplified products were separated and detected on an ABI3500xL Genetic Analyzer with 50 cm capillary array and POP-7 polymer, using injection parameters of 180s at 15 kV (Thermo Fisher Scientific). Prior to amplification with the Investigator HDplex kit, QIAcard FTA punches were washed in nuclease free water. The generated data was interpreted with 3500 Series Data Collection Software v.4 and GeneMapper ID-X version 1.5 (Thermo Fisher Scientific). The analytical thresholds for typing an allele was derived from the laboratory’s current routine case work threshold obtained from internal validation (Supplementary Table S3). The Investigator HDplex, Investigator Argus X12 QS and PowerPlex Y23 kits were amplified and analysed in two separate analysis and the final allele typing was based on concordant alleles from both replicates.

### Sequence-based STR typing

Sequence-based STR typing was performed with the Mainstay SE kit (Qiagen). The preparation of DNA libraries followed the protocol [19], except for the addition and wash of QIAcard FTA punches which is described in [20]. Samples were processed in seven batches, each including one positive control (NA24385) and two negative controls (blank QIAcard FTA punch and nuclease free water, respectively). The number of samples processed in each run varied from 48 to 96 (Supplementary Table S4). QIAcard FTA punches were washed with 1X TBE buffer and a PCR master mix was set up using the enhanced PCR1 buffer system (ePCR1), DNA primer mix D (including SE33), enzyme mix (FEM) and nuclease free water. The reaction was PCR amplified according to the protocol on a Veriti Thermal Cycler (Thermo Fisher Scientific) which was followed by a second amplification for target enrichment and addition of unique dual indexes, utilizing the ProFlex PCR System (Thermo Fisher Scientific). DNA libraries were purified and normalized with magnetic beads. All samples per batch were pooled, denatured and diluted prior to sequencing on a MiSeq FGx (Qiagen) with the Micro reagent kit flow cell. The volume of pooled normalized library loaded for sequencing varied based on evaluation of the first sequencing runs and is detailed in Supplementary Table S4. All other procedures and volumes followed those stated in the protocol. Generated sequencing data was bioinformatically analysed in the Universal Analysis Software (UAS) version 2.5.1 [21]. Alleles were initially assigned using UAS software with default thresholds. To ensure consistency with the recommendations of the DNA commission of the International Society for Forensic Genetics (ISFG) on short tandem repeat sequence nomenclature [22, 23], STRnaming v1.2.0 [24] was used, and concordance between the two software outputs was examined. Final allele frequencies are reported based on the STRnaming analysis. The library file used in STRnaming is provided in Supplementary File S1.

### Quality control

#### Concordance analysis

Five commercial STR kits were used in this study and some STR markers overlapped between the kits (Supplementary Table S5). In total, 40 markers overlapped with at least one other kit and concordance of typed alleles was investigated.

#### Relationship testing

The blind search tool in Familias version 3.3.1 [25] was used to find potential relatives among the selected samples. The tool paired all samples and computed likelihood ratios (LR) for each of the following relationships: direct match, parent-child, siblings, half-siblings, first cousins, and second cousins, all with unrelated as alternative hypothesis. In total 152,076 pairwise kinship comparisons for each hypothesis were made and those with an LR > 1,000 were excluded. This uniform threshold was applied across all tested relationships as a pragmatic and conservative criterion for strong evidential support. As only a limited number of comparisons were affected, further optimization of relationship-specific thresholds was considered unnecessary.

### Population statistics

Allele frequencies for the autosomal STRs together with relevant forensic population parameters, including gene diversity (GD), polymorphism information content (PIC), random match probability (PM), power of discrimination (PD), observed heterozygosity (H_obs_), power of exclusion (PE), and typical paternity index (TPI) were calculated with STRAF [26] in local mode [27] using R software v.4.3.1 [28]. Estimation of Hardy-Weinberg equilibrium (HWE) was assessed with Arlequin v.3.5.2.2 [29] using 1,000,000 steps in Markov chain and 100,000 dememorization (burn-in) steps. Linkage disequilibrium between locus pairs was tested in Arlequin using 10,000 permutations and 2 initial conditions. The level of significance was set to 0.05 but adjusted for multiple testing according to Benjamini-Hochberg (BH) correction [30]. Both the CE and sequence based autosomal STR data were submitted to STRidER [31] for quality control (STR000457). The following population parameters were calculated from the X-chromosomal data using StatsX v2.0 [32]: haplotype diversity (HD), polymorphism information content (PIC), power of discrimination male and female (PDM, PDF), mean exclusion chance in cases of duos and trios (MEC_Krüger_, MEC_Kishida_, MEC_Desmarais_ and MEC_Desmarais duo_). X- and Y-STR haplotype frequencies were determined based on a direct counting method, and X haplotypes were divided in four linkage groups [33]. Haplotype diversity (HD) for the Y-STR haplotypes was computed according to the formula $\:HD=n*\frac{1-\sum\:{{p}_{i}}^{2}}{n-1}$, where *n* denotes the sample size and *p*_*i*_ is the frequency of the *i*-th haplotype. Y-STR haplotype match probability (HMP) was calculated as the sum of squared haplotype frequencies, and discrimination capacity (DC) was computed as the ratio of distinct haplotypes to total number of haplotypes.

Population genetic comparisons were performed separately for the autosomal, X-chromosomal and Y-chromosomal STR datasets. For the autosomal STRs, intra-population analyses were conducted using the CE-based data, first by grouping individuals according to the three major geographical lands in Sweden (Norrland, Svealand and Götaland, Fig. 1), and then at a finer scale across all 21 counties. For both levels of comparisons, analysis of molecular variance (AMOVA), pairwise *F*_*ST*_ estimates, and the Exact test for population differentiation were performed in Arlequin [34] with 10,000 permutations, 100,000 Markov chain steps, and 10,000 dememorization steps. Inter-population comparisons were conducted using data from different STR kits, including geographically close and more distant populations, as well as Swedish reference datasets (Table 1). Pairwise F_*ST*_ estimates and Exact test for population differentiation was performed between the populations. For the X-STRs, intra-population structure was assessed based on the three Swedish lands using AMOVA and pairwise *R*_*ST*_ estimates, calculated separately for each linkage group using Arlequin. The same analysis was extended to include inter-population comparisons with nearby and distant populations (Table 1). Biallelic individuals (*n* = 4) were excluded from the X-chromosomal population comparison. The Y-STR data were analysed similarly in Arlequin, with intra-population comparisons conducted across the three lands of Sweden. AMOVA and pairwise *R*_*ST*_ values were calculated to assess genetic differentiation. These analyses were also extended to additional populations (Table 1) to assess inter-population differences based on Y-STR haplotypes. For simplicity, locus DYS385ab was excluded in the population comparison, as well as two samples with null alleles, two samples with duplicated alleles, and three individuals with intermediate alleles.

**Table 1:** Populations compared for each STR kit and the number of overlapping STR loci between this study and the respective compared population

STR kit (# of STRs)	Population based on country level	Reference	# of overlapping STRs in compared population
PowerPlex Fusion 6 C (23)	Sweden 2008	[35]	15
	Sweden 2011	[36]	15
	Norway	[9]	22
	Switzerland	[37]	23
	Somalia	[38]	15
	Poland	[39]	15
Investigator HDplex (12)	Sweden 2013	[40]	12
	The Netherlands	[41]	
	Switzerland	[5]	
	Poland	[42]	
	Somalia	[40]	
Investigator X12QS (12)	Sweden 2012	[43]	12
	Denmark	[44]	
	Germany	[45]	
	Somalia	[44]	
PowerPlex Y23 (23)	Sweden 2014	[46]	23
	Denmark		
	Finland		
	Germany		
	Poland		
	Switzerland		
	Kenya		

## Results and discussion

### Sample selection

The distribution of samples per county followed the percentual distribution of residents except for Skåne which lacked 5 individuals. Therefore, 5 additional individuals born in the neighbouring county Blekinge were included (Supplementary Table S1). The male-specific sample subset deviated somewhat from the percentual distribution of residents from each county (Supplementary Table S1). This was due to a limited amount of collected male samples from some counties.

### Quality control

Alleles generated with the Investigator HDplex (HDplex), Investigator Argus X12 QS (X12QS) and PowerPlex Y23 (PPY23) kits were based on duplicate PCR amplifications, and the resulting electropherograms were independently interpreted by two individuals. No discordances were observed between replicates. The PowerPlex Fusion 6C (PPF6C) and Mainstay SE data was analysed once, respectively. However, all PPF6C loci and 44 out of 54 Mainstay SE loci overlapped with at least one other kit and final allele calls were based on concordance with an overlapping kit (Supplementary Table S5). For the remaining 10 Mainstay SE loci, for which no concordance data were available, loci flagged with QC indicators were manually re-interpreted by another analyst.

#### Concordance analysis

A genotype concordance rate of 99.96% was observed between the CE-based kits across 2,648 compared genotypes. One discordance was observed in SE33 where PPF6C showed 16/20.2 whereas 16/16 was typed with HDplex, this observation was interpreted as a null allele with HDplex and this sample was therefore excluded from further analysis.

When comparing CE-based and sequence-based genotypes, 11 discordances were identified out of 20,552 genotypes (99.95% genotype concordance). These discordances were manually re-examined by analysing both the electropherograms and sequence data. Of the 11 discordances, six samples analysed with Mainstay SE displayed quality control flag indicators in the UAS. Five of these QC-flagged discordances were attributed to typed stutter artefacts. All these were slightly above the stutter filter and subsequently typed as imbalanced (data not shown). These were manually interpreted and edited according to the CE results. The remaining QC-flagged locus was observed at the same sample and locus as the null allele observed at SE33 with HDplex described above. This locus displayed a significant imbalanced allele ratio (the minor allele was 5% of major allele). This further supported exclusion of this sample. For the five discordances without QC flags, an in-depth analysis of the DNA sequences, including flanking regions, was performed (Table 2). In one sample at the SE33 locus, PPF6C reported alleles 24.2/27.2, whereas Mainstay SE identified 25.2/27.2. Due to its complex sequence motif, SE33 presents challenges in allele nomenclature and discordances have been previously reported [47–49]. The observed discrepancy consisted of the A1 motif pattern and a 3’ flanking region deletion of four nucleotides (CTTC) at positions 88,277,286 to 88,277,289 (GRCh38.p14). Although similar deletions in this region have been reported before [47–49], this specific deletion has, to our knowledge, not been previously published. The four base pair deletion resulted in a fragment being one repeat unit shorter, detected with CE but ignored with UAS nomenclature system. However, when the Mainstay SE data were re-analysed using STRnaming, the resulting allele designation was in concordance with CE (24.2), highlighting nomenclature differences between sequence-based software systems. Notably, SE33 was also included in the HDplex kit which also reported allele 24.2. This particular discrepancy between Mainstay SE and both PPF6C and HDplex was counted as two separate discordances. In another sample, a discordance at the D7S820 locus was observed. PPF6C reported 9.1/10 while UAS typed 9/10 and STRnaming showed concordance with the length-based allele designation (9.1/10). Sequence analysis of the flanking region revealed the alternative allele (nucleotide A) at rs7789995, along with a duplicated A nucleotide in the upstream flanking region (rs1463708262, positions 84160205–84160212, GRCh38.p14). This insertion in the flanking region caused the fragment to be one base pair longer resulting in the point one allele when analysed with CE. This particular variant has been previously reported [50, 51]. Another discordant allele was observed at the PentaD locus. PPF6C typed 9/12.4 while UAS reported 9/13. This discrepancy was due to a downstream deletion at rs536566765 (position 43636277, GRCh38.p14), previously reported in [52], resulting in the one base pair shorter fragment detected with CE. Again, STRnaming was in concordance with the CE nomenclature. The final discordance was observed at DYS19 where allele 14.2 was typed with PPY23 and STRnaming, while UAS reported allele 14. This discrepancy was caused by a TA insertion downstream of the repeat region in a TA block (positions 9684450–9684461, GRCh38.p14), resulting in the point two microvariant allele, similar to findings reported in [53].

**Table 2:** Genotype discordances between CE and MPS data without QC flags in the UAS software

Locus	CE genotype	MPS genotype Mainstay SE UAS	MPS genotype Mainstay SE STRnaming
SE33	24.2/27.2 (PPF6C)	25.2/27.2	24.2/27.2
SE33	24.2/27.2 (HDplex)	25.2/27.2	24.2/27.2
D7S820	9.1/10 (PPF6C)	9/10	9.1/10
PentaD	9/12.4 (PPF6C)	9/13	9/12.4
DYS19	14.2 (PPY23)	14	14.2

Taken together, the reasons for the few observed genotype discrepancies have been identified and are associated to either null alleles, stutter artefacts or inconsistencies in sequence nomenclature software. All mistyped stutter artefacts were flagged with a QC indicator in the UAS, and manual data interpretation would prevent incorrect allele typing. The CE-based kits investigated in this study have been internally validated and implemented in routine casework at the laboratory whereas Mainstay SE has been internally validated but not yet implemented in routine casework. These findings therefore emphasize the importance of thorough validation and the definition of appropriate allele calling thresholds prior to implementation. Understanding discrepancies between CE and sequencing data is crucial to facilitate the integration of STR sequencing in forensic genetics. Compatibility with national and international DNA databases is essential. As demonstrated here, this version of the UAS software has limitations in allele designation since only the repeat units are accounted for when assigning nomenclature. As shown, variants in the flanking region affects the complete fragment length and must to be considered to ensure compatibility with traditional length-based nomenclature. Similar findings have been previously reported by several studies [12, 50, 54–59] and multiple causes for CE-MPS discrepancies have been identified such as; different primer positions causing different amplicon sizes, variants in the primer binding site, INDELS in the flanking regions and differences in the sequence analysis range by different software [50]. Complete nomenclature concordance with CE was, however, observed when using STRnaming for allele naming which also is the recommended software by ISFG for STR sequencing nomenclature [23].

#### Genotyping anomalies

Marker D21S2055 in the HDplex kit exhibited consistently imbalanced peak heights in heterozygous genotypes across multiple samples, complicating the reliable differentiation between true alleles and stutter peaks. Due to these interpretation challenges, this marker was excluded from all subsequent analysis. Similar imbalances have also been reported in previous studies [5, 40].

Locus D18S51 displayed a tri-allelic pattern in one individual across all three kits (HDplex, PPF6C, and Mainstay SE). Alleles 19, 21 and 22 were observed with uneven peak heights, where the sum of the height of allele 21 and 22 was close to the height of allele 19. This pattern represents a typical “Type I” tri-allelic pattern [60], which mainly are caused by somatic mutation early in the cell development. Similar tri-allelic patterns at different STR locus have been reported by several others [61–64]. Furthermore, D18S51 is the most frequently reported locus displaying three alleles at the Short Tandem Repeat DNA Database (STRbase v2.0) [65], however, this specific combination of alleles has not yet been reported in the database. This sample was excluded from all subsequent analysis.

Three samples displayed three alleles at locus D17S1301, included in the Mainstay SE kit. This observation has not been previously reported in STRbase nor published, according to our knowledge. These samples were excluded in the final dataset from which allele frequencies were estimated. The Y-STRs included in Mainstay SE were only subject for concordance analysis with PPY23 and has not been further analysed. Due to the smaller MPS-typed male subset (*n* = 248), comprehensive sequence-based evaluation was considered beyond the scope of the present study.

From the analysis of the X12QS kit, a single allele per locus was expected since only male samples were analysed. However, biallelic pattern was observed in four samples at four distinct loci (DXS10079, DXS10135, DXS10146 and DXS8378). This type of biallelic patterns of the X-chromosome have been previously reported at several loci [66–72]. Biallelic pattern was also observed from the PPY23 data in two samples at two separate loci (DYS635 and DYS439). Both these loci and the same allelic combination has been observed in other studies [46] and reported to STRbase [65]. Two samples displayed null alleles at one locus (DYS391). Of the 516 males typed with PPY23, 248 were also analysed with Mainstay SE. One of the two null allele samples belonged to this subset and showed allele 10 with Mainstay SE. This further supports that this is a true null allele observed with PPY23 and not caused by a technical issue. None of the biallelic or null alleles observed at X-STR or Y-STR loci were excluded from the final population set. Since all individuals in the dataset were males, genotyping results were interpreted as haplotypes where the full combination of alleles across loci, rather than individual alleles, are considered. These findings were retained to preserve the haplotype structure and to reflect the actual diversity present in the dataset.

#### Relationship testing

Pairwise kinship comparisons using the blind search module in Familias revealed 9 pairs with an LR > 1,000 for at least one of the tested hypotheses (data not shown). Therefore, nine individuals were excluded from the autosomal dataset. Three of these pairs of assumed relation were males, wherefore three individuals from the male specific subset were excluded from further analysis.

### Population genetic statistics

#### Autosomal CE-based data

The final autosomal dataset consisted of 538 individuals from which allele frequencies were estimated at all PPF6C and the 11 remaining HDplex loci, resulting in 31 STRs (Supplementary Table S6). Test of Hardy-Weinberg equilibrium (HWE) revealed significant p-value (*p* < 0.05) in one locus (D10S1248). However, no indication of deviation from HWE was observed when accounting for multiple testing applying BH correction (Supplementary Table S6). Twenty locus pairs displayed significant linkage disequilibrium (*p* < 0.05), however, none remained significant after applying adjusted p-values for multiple testing according to BH (Supplementary Table S7). All statistical parameters presented in Supplementary Table S6 indicated that TPOX was the least informative marker, whilst SE33 was the most informative.

#### Autosomal sequence-based data

**Fig. 2:**
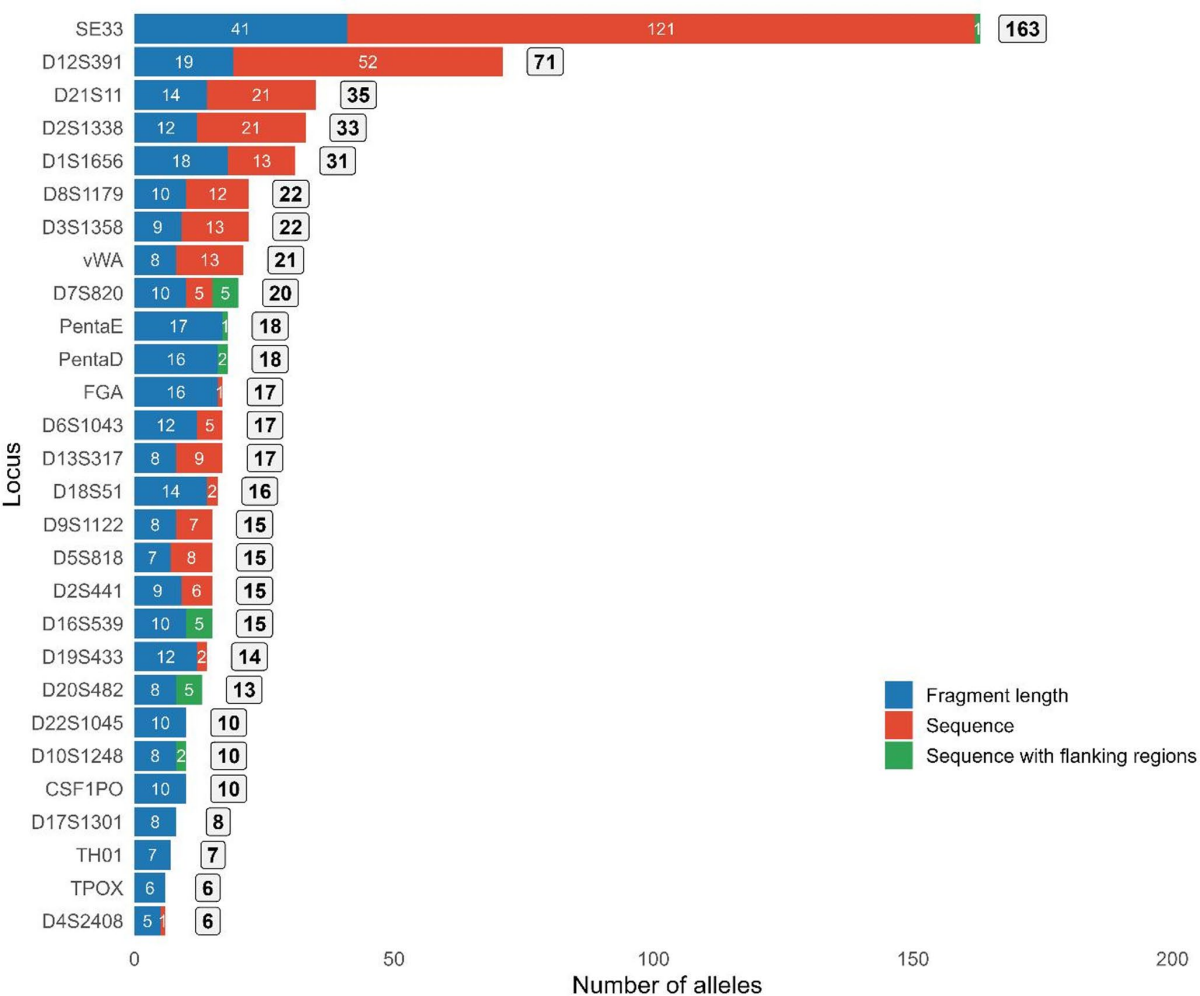
Number of observed alleles at autosomal loci with mainstay SE based on fragment length (blue), sequence (red), and sequence with flanking regions (green). The number to the right of each bar represent the total number of alleles

Sequence-based alleles were generated with Mainstay SE based on the same 538 individuals. Alleles were assigned using STRnaming, which captures sequence variation in both repeat- and flanking regions. Allele frequencies were calculated both from traditional repeat lengths, comparable to CE, and from complete sequences, thereby accounting for variation within the repeat motif and adjacent flanking regions. Length-based and sequence-based allele frequencies together with the full sequence is reported in Supplementary Table S8. Consistently with the CE-based findings, TPOX and SE33 displayed the lowest and highest variation, respectively, across all investigated population genetic parameters (Supplementary Table S9). The number of alleles per locus increased with sequencing at 23 of the 28 examined loci, with SE33 displaying the largest variation from 41 to 163 alleles (Fig. 2). The total number of observed sequence-based allele variants was 665 compared to 332 length-based, reflecting a twofold increase. This overall trend is consistent with findings in other Scandinavian populations [6, 9] and in a previous study of Swedes [12]. However, the magnitude of the increase differed, as the previous Swedish study [12] reported a 55% increase in allele observations. This discrepancy may be attributed to the inclusion of over 140 additional individuals analysed in this study and including the highly polymorphic locus SE33, as well as the consideration of flanking region variants, which were not analysed previously. As shown in Fig. 2, sequence-based differences within the repeat region accounted for most of the increase, whereas flanking region variants were comparatively few. The increase was concentrated at a few loci, and the median increase in the number of observed alleles when using MPS across all loci was five. Although the total increase of alleles in the population was substantial, the overall frequency was considerably low for some variants. For comparison, the number of alleles below the commonly used 5/2 N frequency (0.46% in this study) [73] was 314 with sequence-based analysis and 84 with the length-based approach, resulting in a 274% increase in low frequency variants. This highlights that the relative increase in low frequency variants exceeds the overall allele count increase. This observation aligns with previous findings [12] suggesting that the overall impact on the weight of evidence at the population level may be limited for kinship and identity testing. Nevertheless, sequence information remains informative and powerful in specific cases, as it captures underlying genetic diversity.

Test of HWE for all loci was performed for length-based and sequence-based data independently, and no significant deviation (*p* < 0.05) was observed in either dataset using BH correction (Supplementary Table S9). One locus (D10S1248) in the length based, and two loci (D20S482 and D2S441) from the sequence-based dataset did, however, display significant raw p-values. Pairwise test of linkage disequilibrium across all 28 loci (378 comparisons) showed 21 and 26 significant p-values (*p* < 0.05) for length-based and sequence-based data, respectively. No significant p-values were, however, obtained after BH correction (Supplementary Table S10). The combined random match probability (PM) was 9.34E-34 for length-based and 3.48E-38 for sequence-based data. These numbers are similar with other studies [6, 55], although slightly higher which may be due to including SE33 in this study. As discussed by Hussing et al. [6], the increase in random match probability is relatively limited compared to the cost of implementing STR sequencing in casework. However as previously discussed, increased genetic diversity may still provide meaningful insights in individual cases.

#### Sex-chromosomal data

The final male-specific population dataset consisted of 513 individuals, following the exclusion of three potentially related individuals. X-chromosomal STR analysis revealed entirely unique haplotypes for all individuals. Haplotype frequencies per linkage group (LG) is shown in Supplementary Table S11, S12, S13 and S14. Forensically relevant X-chromosomal population genetic parameters are presented in Supplementary Table S15, all indicating high informative value in the Swedish population, confirming suitability of these markers in forensic identification and kinship cases. Linkage Group 1 was found to be the most polymorphic group with 315 different haplotypes, including 210 singletons, while LG3 displayed least variation, similar to the previous study of Swedish [43] and other European populations [72, 74]. Pairwise linkage disequilibrium analysis was performed for all loci. Association was observed between loci within each LG when applying BH adjusted p-values. No significant p-values (*p* < 0.05) were, however, observed between loci from different LGs (Supplementary Table S16).

A total of 508 different Y-STR haplotypes were identified among the 513 analysed male samples (Supplementary Table S17). Of these, 503 were only observed once (98.0%) and the remaining 5 haplotypes were each observed twice. Individuals that shared Y-STR haplotypes were analysed with autosomal STRs and included in the relationship testing described in the Materials and Methods section. No indication of relationship among these individuals were observed. The haplotype diversity was 0.99996, indicating an exceptionally high level of male-specific genetic variation. The haplotype match probability was 0.002, and the discrimination capacity was 99.0%, reflecting the high individualizing power of the Y-STR marker set. These values are comparable to those reported in other European populations [46], and demonstrates the robustness of the selected loci in differentiating unrelated males. Overall, the findings confirm the suitability of the PowerPlex Y23 markers for forensic applications, including kinship analysis, in the Swedish population.

### Population comparison

#### Autosomal intra-population analysis

To assess regional genetic structure within Sweden, an AMOVA was performed using STR data across all 31 loci in PPF6C and HDplex kits. Individuals were grouped into three major geographic lands of Sweden (Norrland, Svealand, and Götaland). The analysis revealed that differences among lands accounted for only 0.05% of the total genetic variation, while the majority (99.95%) was observed within lands (Table 3). The global *F*_*ST*_ was very low (0.0005, p-value = 0.03), indicating minimal population substructure at the regional level. Pairwise *F*_*ST*_ comparisons supported this conclusion, with all values falling below 0.00067 (Supplementary Table S18). The only statistically significant differentiation was observed between Svealand and Götaland (*F*_*ST*_ = 0.00067, *p* = 0.007, BH-adjusted *p* = 0.022), however, the magnitude of this differentiation was very low and unlikely to represent a meaningful population division. Furthermore, Exact test of population differentiation yielded non-significant results for all pairwise comparisons, further confirming the lack of detectable regional substructure (Supplementary Table S19). For a more fine-scale analysis, individuals were grouped according to Sweden’s 21 counties. A similar distribution of genetic variation was observed in the AMOVA results (Table 3), with nearly all genetic variation observed within counties rather than between them. This pattern was further illustrated by MDS plot and a heatmap of average pairwise *F*_*ST*_ values across all loci (Supplementary Fig. S1). With the exception of two comparisons between Västmanland-Örebro (*F*_*ST*_ = 0.016, *p* = 0.021), and Västerbotten-Kalmar (*F*_*ST*_ = 0.015, *p* = 0.021), all pairwise *F*_*ST*_ values were non-significant, and the median *F*_*ST*_ value across all county comparisons was 0.0019. Notably, Gotland displayed a somewhat elevated average *F*_*ST*_ of up to 0.03 (Supplementary Table S18). Per-locus *F*_*ST*_ analysis (Supplementary Table S20) revealed that this increase was driven by a few loci with high *F*_*ST*_ values, while most loci showed no differentiation. The sample size from Gotland (only three individuals, Supplementary Table S1) likely contributed to this effect, as the presence of rare alleles in such a limited dataset can inflate *F*_*ST*_ values and overestimate genetic differences. The overall pattern of genetic differentiation observed in this study was consistent with findings from a previous Swedish study using 34 autosomal SNPs [75]. However, genome-wide SNP studies [76, 77] generally reported even lower *F*_*ST*_ values between Swedish counties than found in this study. This difference is somewhat expected as STRs have higher mutation rates and greater allelic diversity than SNPs, which can result in elevated estimates of genetic differentiation. Additionally, the relatively small number of STR loci used here, compared to the dense marker coverage in genome-wide SNP datasets, may increase the effect of local substructure. Moreover, our limited sample sizes at the county level may have introduced sampling effects, and thus, interpretation at the county level should be made with caution. Importantly, when individuals were grouped into the three major geographic lands, where sample sizes were larger, the *F*_*ST*_ values were more similar to those reported in the genome-wide studies [76, 77]. Taken together, these results indicate that genetic structure within Sweden is minimal, based on the STR data analysed here. These findings support the use of a single, unified Swedish reference population for forensic population genetic analyses.

**Table 3:** AMOVA across 31 loci from the PPF6C and HDplex kits based on grouping into three lands and 21 counties of Sweden, respectively

Group	Source of variation	% of total variation
The 3 lands of Sweden	Among lands	0.05
	Within lands	99.95
The 21 counties of Sweden	Among counties	0.12
	Within counties	99.88

#### Sex-chromosomal intra-population analysis

Intra-population analysis of the X-chromosomal markers were based on haplotypes from the different linkage groups, divided in the three lands (Norrland, Svealand, and Götaland). The AMOVA revealed minimal genetic differentiation among the lands for all linkage groups (Supplementary Table S21). Pairwise *R*_*ST*_ values further confirmed this since no statistically significant differentiation between any of the regional pairs were detected, and all *R*_*ST*_ values were < 0.008 (Supplementary Table S22). These results indicate a lack of significant regional population substructure based on X-chromosomal data.

An AMOVA was performed based on Y-STR haplotypes divided in Norrland, Svealand and Götaland together with another dataset of a Swedish population (“Sweden 2014”) from [46]. The grouping was structured into two major groups: this study and Sweden 2014. The AMOVA revealed minimal genetic differentiation among the lands (Supplementary Table S23). Comparisons between Norrland, Svealand and Götaland from this study showed low *R*_*ST*_ values (0.005–0.01), suggesting limited but measurable regional structure within Sweden.

#### Autosomal inter-population analysis

**Fig. 3:**
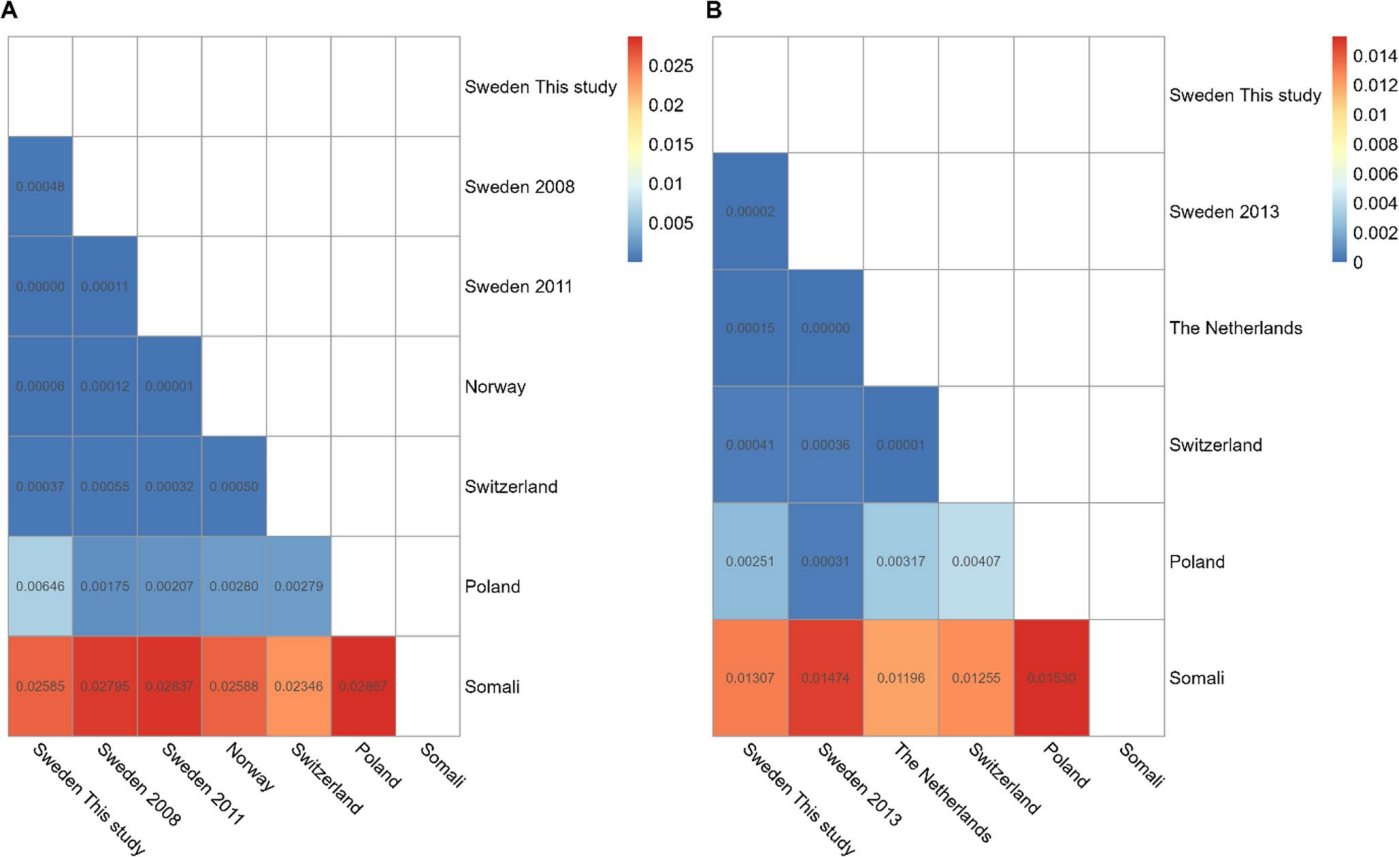
Pairwise *F*_*ST*_ values between different populations are illustrated as heatmaps calculated from PPF6C (**A)** and HDplex (**B**) loci. Different colours reflect the magnitude of genetic differentiation in each pair

Genetic differences were investigated between CE-derived autosomal data from this study with both currently applied Swedish allele frequencies and geographically close and distant populations based on allele frequencies. Population comparisons of the autosomal STR markers were divided in two sets, representing the PPF6C and HDplex loci. Three separate allele frequency databases representing a Swedish population, with different underlying data, are currently used in Sweden, hereafter referred to as “Sweden 2008” [35], “Sweden 2011” [36], and “Sweden 2013” [40]. Both the “Sweden 2008” and “Sweden 2011” dataset overlap with 15 of the PPF6C markers, although not the same 15. All HDplex markers overlap the data presented in “Sweden 2013”. PPF6C data was compared with the two Swedish data sets [35, 36], three European populations (Norway [9], Poland [39], and Switzerland [37]), and a Somali population [38]. The HDplex data was compared with the Swedish (“Sweden 2013” [40]), three European populations (The Netherlands [41], Poland [42], and Switzerland [37]), and a Somali population [40]. An Exact test of population differentiation per locus, and average *F*_*ST*_ estimates were calculated pairwise between the Swedish population presented in this study and all other populations using Arlequin. Pairwise average *F*_*ST*_ values with compared populations (Fig. 3) revealed minimal genetic differentiation between this study and the other Nordic and Central European populations (*F*_*ST*_ < 0.0005), indicating high genetic similarity. As expected, genetic differentiation with the Somali individuals was notably higher (0.013 and 0.026 for HDplex and PPF6C, respectively), reflecting the geographical distance. Interestingly, the Polish population showed slightly higher *F*_*ST*_ (0.006 (PPF6C) and 0.002 (HDplex)) compared to the Nordic and Central European populations. The Exact test for population differentiation revealed similar findings (Supplementary Table S25). Significant p-values (*p* < 0.05) after BH correction for multiple testing was observed at all except three loci in the Somali population and at four loci in the Polish population. No other comparison indicated significant population differentiation. These observations align with previous findings of the Swedish population [35, 40].

#### Sex-chromosomal inter-population analysis

The X-chromosomal population data was compared with data from four different populations from Sweden [43], Denmark [44], Germany [45], and Somali [44]. From the AMOVA, a majority of the genetic variation was found within these populations, while variation among the populations ranged from 0.49% to 5.78% for the different linkage groups (Supplementary Table S21). Minimal genetic differences were observed between the Swedish, Danish, and German populations, respectively. Low pairwise *R*_*ST*_ values (Supplementary Table S26) were obtained between the two Swedish datasets (< 0.003) and between Sweden and the two other European populations (< 0.01), all non-significant. All except one p-value for *R*_*ST*_ estimates between Sweden and the Somali population were, however, significant (*p* < 0.05) and the *R*_*ST*_ ranged from 0.015 to 0.20. These observations align with expected differences due to geographical locations and follows observations from other studies [43, 44, 74].

For the Y-STRs, the AMOVA revealed significant genetic structure among the investigated populations (Supplementary Table S23). Seven predefined groups were examined (Sweden, Denmark, Finland, Germany, Poland, Switzerland, and Kenya). The majority of the molecular variance was found within these populations (90.87%), while 8.59% variation was observed among the groups. Variation among populations within the Swedish group accounted for 0.54%. Furthermore, the pairwise *R*_*ST*_ values (Supplementary Table S27) highlighted substantial differentiation between populations, particularly between Kenya and the other European populations with *R*_*ST*_ values ranging from 0.14 to 0.20, all statistically significant after BH correction. The Swedish group consisted of two subsets, combined Swedish haplotypes from this study and the combined Swedish dataset from [46]. These two Swedish datasets showed minimal genetic divergence (*R*_*ST*_ = 0.006), as expected for samples from the same region. Slightly higher *R*_*ST*_ values were observed between Sweden and Denmark, Germany and Switzerland, respectively (*R*_*ST*_ 0.02–0.07). In contrast, Finland exhibited higher divergence from other Nordic populations (0.13–0.15), which is consistent with previous studies identifying the Finnish population as a genetic outlier in Europe [78, 79].

## Conclusions

We have systematically collected blood samples from all counties in Sweden and performed DNA typing using five forensic STR kits targeting autosomal, X- and Y-STRs, applying both traditional capillary electrophoresis and massively parallel sequencing. Consequently, both length-based and sequence-based allele frequencies are presented. The analysed STR markers demonstrated exceptionally high discrimination power across all forensic population genetic parameters, confirming their suitability for forensic applications in the Swedish population. A high concordance rate (> 99.95%) was observed between all investigated STR kits with only a few discordances, primarily caused by differences in nomenclature between CE and MPS generated data. Significantly low overall genetic differences were observed within Sweden (0.05% and 0.12% variation between lands and counties, respectively). Only minor genetic differences were found between Sweden and other Nordic and central European populations. As expected, significant population differentiation was found between populations geographically distant to Sweden, underscoring the importance of accounting for population structure when choosing reference datasets. This work has resulted in an updated and expanded reference database representative of the contemporary Swedish population, which is expected to serve as a valuable resource for forensic casework in Sweden.

## Supplementary Information

Below is the link to the electronic supplementary material.

Supplementary Material 1 (TXT 2.52 KB)

Supplementary Material 2 (DOCX 660 KB)

Supplementary Material 3 (XLSX 530 KB)
