# Supplementary material for: Swedish population data for 37 autosomal, 12 X-chromosomal and 23 Y-chromosomal STR loci
Source: Int J Legal Med. 2026 Apr 9;140(4):2041–54. doi: 10.1007/s00414-026-03793-2 (PMC13275542; doi:10.1007/s00414-026-03793-2)
Supplement: Supplementary file 2 — Supplementary Material 2 (DOCX 660 KB) [file 414_2026_3793_MOESM2_ESM.docx]

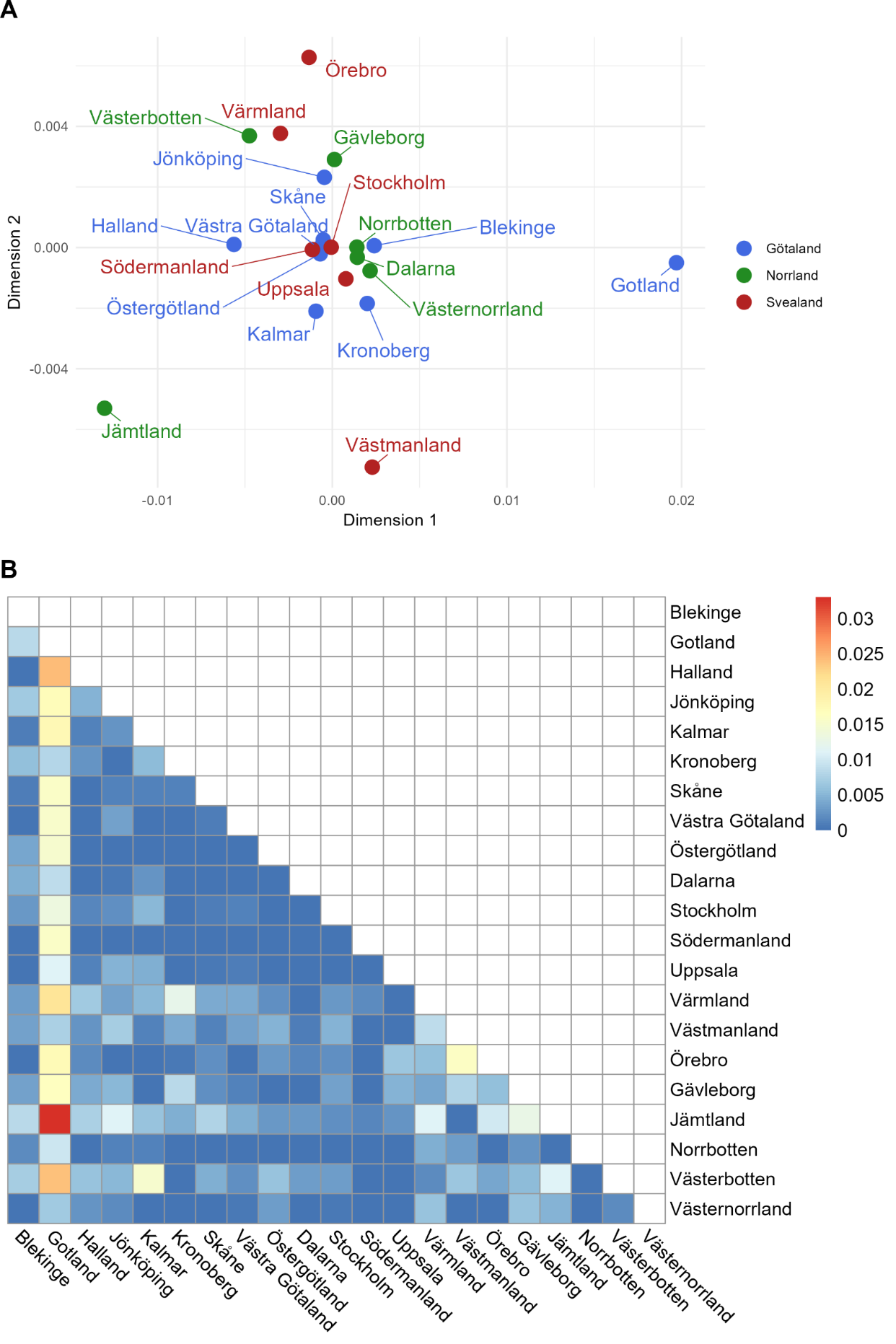


**Supplementary Fig. S1** Pairwise *F_ST_* values between each of the 21 counties in Sweden calculated from autosomal STR data (PPF6C and HDplex loci). A) Multidimensional scaling (MDS) plot. Each point represents a county, positioned based on genetic distances derived from *F_ST_*. The counties are coloured by the three lands of Sweden. B) Heatmap with different colours reflecting the magnitude of genetic differentiation in each pair
